# Analysis of Intestinal Injuries Induced by Ricin *in Vitro* Using SPR Technology and MS Identification

**DOI:** 10.3390/ijms10052431

**Published:** 2009-05-22

**Authors:** Linna Liu, Hongwei Gao, Jiping Li, Ying Dong, Ning Liu, Jiayu Wan, Wensen Liu, Yucheng Sun, Ming Xu

**Affiliations:** 1 Institute of Military Veterinary, Academy of Military Medical Science, Changchun 130062, China; E-Mails: liulinna7@126.com (L.L.); lijiping66@126.com (J.-P.L.); lws0901@126.com (W.-S.L.); dongying0918@126.com (Y.D.); sunyuchengsyc@126.com (Y.-C.S.); wanjiayu@126.com (J.-Y.W.); xumingjlu@126.com (M.X.); 2 Department of Theoretic Veterinary Medicine, Colledge of Animal Science and Veterinary, Jilin University, Changchun 130062, China

**Keywords:** ricin, BBM, SPR, HPCE-ESI-MS

## Abstract

The present study found that ricin toxicity did not only manifest itself as inhibition of protein synthesis, but also induced apoptosis of immune cells and played an extremely significant role in intestinal injury. In this report, we describe a novel method to estimate binding events occurring on intestinal brush border membranes (BBM) based on SPR technology in an attempt to mimic the real intestinal surface capable of interacting physically and/or actively with certain biological molecules. Combined with HPCE-ESI-MS indentification, we obtained 28 kinds of proteins in BBM that interacted with ricin.

## 1. Introduction

Upon passive and/or active transport, most toxins or drugs developed for oral administration are first absorbed in the gastrointestinal tract, followed by systemic circulation. Because *in vivo* investigations are, however, time-consuming and it is often difficult to quantify the results, it is evident that the predicative *in vitro* model is needed.

The surface plasmon resonance (SPR) biosensor, based on the detection of a refractive index change on a gold surface, has been widely used to investigate binding events occurring on biological surfaces. [1,2]. Without any need to label the ligands or complex sample preparation, SPR rapidly offers valuable information on the rate and extent of adsorption, association/dissociation kinetics, and the affinity constants of specific ligand-receptor interactions [3,4]. In addition, SPR is one of the few applicable techniques that allows monitoring dynamic interactions within a fluid environment that is similar to conditions encountered *in vivo*.

Ricin was classified as a Category B priority pathogen by the Centers for Disease Control and Prevention (CDC) and is one of the most toxic biologic agents known. Ricin might be delivered by a variety of routes, including injection, ingestion (contaminated food and water), and inhalation (exposure to aerosols) — all routes that pose major threats from a bioterrorism perspective. Derived from the bean of the castor plant, *Ricinus communis*, the toxin is relatively easy to produce in massive quantities at minimal cost in a low-technology environment. Ricin is an example of a multi-chain ribosome-inactivating protein (RIP) toxin. These toxins inhibited protein synthesis, preventing new growth and leading to cell death [5].

In the present study, we describe a novel method to estimate binding events occurring on intestinal membranes based on the SPR technology. In an attempt to mimic the real intestinal surface capable of interacting physically and/or actively with ricin, we attached BBMP, isolated from Sprague–Dawley (SD) rats, on the surface of the sensor chip composed of dextran matrix modified with lipophilic residues and also indentified the components that interacted with ricin by CE-ESI-MS.

## 2. Experimental Section

### 2.1. Materials

The SPR instrument was a Biacore 3,000 (Biacore AB, Uppsala, Sweden), equipped with a CM5 sensor chip. Sensor Chip CM5 (research grade), HBS-EP buffer pH 7.4 (10 mM 4-[2-hydroxyethyl] piperazine-1-ethane-sulphonic acid (HEPEs), 150 mM-NaCl, 3 mM EDTA, 0.005% (v/v) surfactant P-20), ready-to-use 10 mM sodium acetate (pH 5.0) and amine coupling kit (400 mM *N*-ethyl-N’-(3-ethylaminopropyl) carbodiimide hydrochloride (EDC), 100 mMN-hydroxysuccinimide (NHS) and 1 M ethanolamine hydrochloride) were obtained from Biacore AB (Uppsala, Sweden). Pure ricin (purity ≥ 95%) was supplied by the biotoxin lab of the Military Veterinary Institute. All other chemicals were purchased from Sigma Chemical Co. (St. Louis, MO) or other commercial sources at the highest purity available.

### 2.2. Brush Border Membrane Protein (BBMP) preparation [6]

For the preparation of BBMP, small intestines were obtained from SD rats (200 – 220 g). Briefly, a homogenate of the mucosa scraped with a glass slide was prepared in buffer A (2 mM Tris-HCl, 50 mM mannitol, PH 7.1). Thereafter CaCl_2_ was added to give a concentration of 10 mM, allowed to stand at 4 °C for 20 min, and centrifuged again at 27,000 g for 30 min. The pellet obtained was washed twice with buffer B (10mM Tris-HCl buffer, 300 mM mannitol, PH 7.1).

### 2.3. Preparation of biosensor chips

BBMP was immobilized on the carboxymethylated dextran layer on the sensor surface of a CM5 chip using the amine coupling kit in combination with the surface preparation wizard as present in the Biacore 3,000 control software. In short, the biosensor surface was activated by injecting at a flow of 5 μL min^−1^) a mixture of EDC and NHS (1:1 v/v, 35 μL) into a flow channel (Fc). BBMP diluted to 100 μg mL^−1^ in coupling buffer (10 mM sodium acetate ; pH 5.0) was injected over the activated surface (immobilised BBMP onto Fc2 with Fc1 as referance). After coupling, active groups were blocked by injecting ethanolamine (1 M) for 7 min at 5 μL min^−1^.

### 2.4. Kinetics analysis

The reference and detection Fcs were connected in series and the response of the referance Fc was subtracted from the response in the detection Fc. In the final format, different concentraions of ricin (0, 1, 5, 10, 20 μM) diluted with HBS-EP buffer in a microtiter plate and used “kinetics analysis” wizard to continue the detection with HBS-EP as the running buffer. Regeneration was performed with 20 μL of 50 mM NaOH at a flow of 20 μL min^−1^, temperature was kept at 25 °C The relative responses used for calculations were measured 5 s before the regeneration was about 7 min.

### 2.5. Mass-Spectrum identification

This work was performed by the Biochemical Analysis group of the Proteome Research Center, Chinese Academy of Sciences. The instrument used in the experiment was a HPCE-ESI-MS (Termo Finnigan, San Jose, CA). Raw files were searched in ipi Mouse v3.36 protein bank with the BIOWORKS software.

## 3. Results and Discussion

### 3.1. Immobilisation of BBMPs on the surface of sensor chip

**Figure 1.**
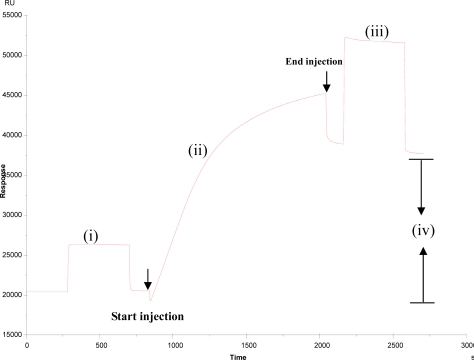
Formation of BBMP surface on the sensor chip. The BBMP suspension was injected at a flow rate of 5 μL min^−1^ over the sensor chip, which had been conditioned with CHAPS, until a response unit value reached 6,000 – 8,000. Excess BBMPs were removed by washing with a HBS-EP buffer for at least 2 h. once stabilized, free BBMP surface was readily regenerated by 50 mM NaOH. The resulting BBMP surface was very stable with a drift < 0.2 RU/min. Arrows represent the beginning and end of each injection. (i) Activation by NHS and EDC. (ii) Immobilization process. (iii) Blocked by injecting ethanolamine. (iv) RU of BBMP immobilized on the chip (about 17,000 RU).

For the formation of the real intestinal membrane on the SPR sensor chip, the BBMPs that were obtained from SD rats were passed over the surface of the CM5 chip containing dextran derivatives modified with lipophilic residues. A typical SPR sensorgram for the BBMPs immobilization on the chip was shown in Figure 1. Because BBMPs were not covalently attached to the CM5 chip, but rather were immobilized by physical interactions, it was of paramount importance to find conditions capable of retaining BBMPs without any significant loss. The results showed that after the injection of the BBMP suspension, the SPR signal was stabilized at an almost constant level of 17,000 RU. The deviation of stable response level might appear due to the flexability and roughness of the dextran matrix that possesses a larger surface area than a flat surface [7].

For the repeated use of the BBMP surface for SPR analysis, we attempted to find conditions under which reconstruction of the BBMP surface was achieved throughout the dissociation of bound analytes without any significant deterioration. It should be noted that the regeneration of the BBMP surface by 50 mM NaOH was not achieved until the RU value reached the stable level. This stability for repeated use was comparable with the results obtained by Cooper *et al*. [8], who prepared supported phospholipid monolayers on the hydrophobic sensor chip, forming an octadecane-thiol self assembled monolayer on a gold surface.

### 3.2. Kinetics analysis of BBMP and ricin

**Figure 2.**
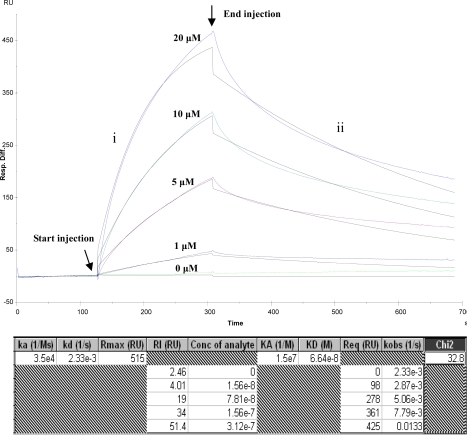
Kinetics analysis of BBMP and ricin. Five different concentrations of ricin were injected over the BBMP bound to the sensor chip at a flow rate of 20 μL·min^−1^ in the HBS-EP buffer: (a) 0 μM; (b) 1 μM; (c) 5 μM; (d) 10 μM; (e) 20 μM. Arrows represent the beginning and end of each injection. i. Association process; ii. Disassociation process.

The binding capacity of BBMPs attached on the chip with ricin was estimated by flowing different concentrations of ricin at a rate of 20 μL·min^−1^(Figure 2). Upon the injection of ricin, the SPR signal rapidly increased by the association with BBMPs, followed by a slower increase to reach equilibrium. After the end of injection, the replaced running buffer dissociated bound ricin. The SPR signal correlated well with the initial ricin concentration, indicating that the amount of ricin bound to BBMP was dependent on the concentration. The binding constant (KA) was 1.5 × 10^7^ and dissociation constant (KD) was 6.64 × 10^−8^, Chi < 1/10 Rmax, supports at the credibility of the framework. This result obviously suggests a specific interaction of ricin with BBMPs. Along this line of research, the detailed binding affinity and kinetic for different toxins or drugs for oral administration are currently under investigation. To date, small intestinal brush border membrane proteins (BBMPs), prepared from the small intestinal mucosa, are gaining recognition as an alternative tool to investigate the active transport because they contain phospholipids, hydrolytic enzymes, and carrier proteins that are responsible for many biologic binding events [9,10]. In particular, BBMPs can be used to estimate the absorption of drugs and nutrients that may be orally administrated because of their structural and functional similarity to real intestinal membrane [11,12].

### 3.3. Recovery and identification of interaction components

**Figure 3.**
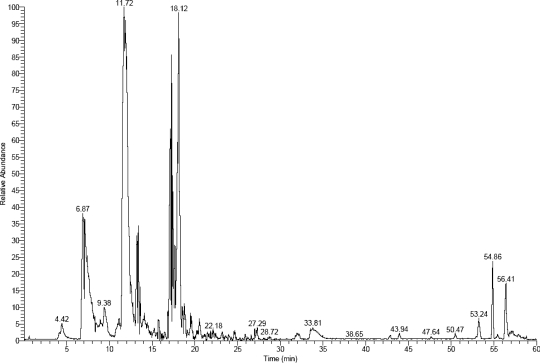
Interaction components seperation by capillary electrophoresis. Seperation column: 0.15 mm × 150 mm (RP-C18) (Column Technology Inc.); Seperation time: 60 min; Abscissa presents the retention time of every protein peak and one peak may contain several components, so after seperation, it was necessary to further indentify the components by mass spectrometry.

A SPR External Recovery Unit was used to recover interaction components of BBMPs with ricin. In order to obtain the recovered components, we immobilized ricin (100 μg/mL) on the whole chip without differentiating channels as much as possible (general 30,000 – 40,000 RU), and BBMP as mobile phase which was convenient for recovery. Procedures were similar to the kinetics analysis: coupling ricin 20 minutes, flow rate 5 μL/min and 50 mM sodium hydroxide as regeneration buffer. After washing the chips with HBS-EP buffer for 2 h, and diluting BBMP with the same buffer the recovery process began. Then, recovered components were indentified by HPCE-ESI-MS (Figure 3).

Finally, we identified 28 kinds of proteins that interacted with ricin (Table 1). They could be broadly classified into five categories, 1) hydrolases; 2) neural regulation proteins; 3) transport-related proteins; 4) nucleoproteins; 5) cytoactive regulation proteins. The functions of some proteins are unkown up to now. Researchers had reported [13,14] that the toxic effect of ricin was correlated with KDEL sequence and vacuolar protein, which had also been detected in our experiment, but the subsequent mechanism remains unknown. These results not only provid new evidence and a research window for the mechanism of intestinal injury induced by ricin, but also set up a new platform to clarify the specific toxic effect of ricin.

## 4. Conclusions

In the present study, we have analyzed the intestinal injury induced by ricin *in vitro*, based on the technology platform of SPR and mass spectrometry, and identified 28 kinds of proteins in BBMPs that interacted with ricin. This detection method compensated for the lack of quantitative analysis *in vivo* when the systemic circulation of toxins or drugs is evaluated and shoul supply much more additional information about the transduction of toxins and drug delivery in the gastrointestinal tract. Nevertheless, in this we might lose some proteins present in low amounts, so in the subsequent research steps, we will strive to improve the detection method in order to obtain a full protein profile of the interaction of BBMPs with ricin. Additionally, will further verify the interaction between ricin and every protein which had been indentified and study in depth their structure and biological function based on proteomics technologies.

## Figures and Tables

**Table 1.** Identification of BBMP components interacting with ricin. After zymohydrolysis, the peptide sections were extracted and the data collected by ESI-MS. BIOWORKS software was used to match the raw files in the ipi MOUSE v3.36 protein bank.

**NO.**	**Cover Percent (%)**	**MW(D)**	**PI**	**Identified Name**
1	1.27	208984.41	5.7	sucrase-isomaltase
2	2.35	112982.21	5.3	Na+/K+ -ATPase alpha 1 subunit
3	8.13	26134.64	4.75	protease, serine, 1
4	1.18	87436.93	5.96	dipeptidylpeptidase 4
5	2.82	81375.48	7.31	Tripartite motif-containing protein 71
6	1.24	109651.12	5.62	alanyl (membrane) aminopeptidase
7	0.92	142634.26	4.26	protocadherin 24
8	0.95	103676.35	5.49	WD repeat domain 22
9	4.67	25110.87	9.14	KDEL (Lys-Asp-Glu-Leu) ER protein retention receptor 3
10	1.28	79500.51	5.59	meprin 1 beta
11	1.57	71901.5	4.76	Rab11-FIP4-like
12	0.42	242072.22	6.54	BAP28 protein
13	0.32	278603.97	6.27	X-linked nuclear protein
14	2.67	43129.22	5.27	guanylate binding protein 8
15	3.13	35989.95	5.43	annexin A4
16	11.65	11368.23	11.18	histone 4 protein
17	2.37	49225.49	5.79	secretion regulating guanine nucleotide exchange factor
18	0.93	105319.96	5.6	similar to neurofilament protein
19	0.3	369730.12	4.97	URE-binding protein 1
20	2.28	81229.48	9.28	junctophilin 3
21	0.25	413981.02	5.46	similar to maltase-glucoamylase
22	0.57	221497.62	5.69	Myosin cardiac muscle beta chain
23	0.27	490969.47	6.11	vacuolar protein sorting 13 D
24	4.41	40820.65	5.32	t-complex protein
25	7.20	13591.96	9.21	Chemokine-like protein TAFA-5.
26	3.48	32599.11	5.95	annexin A2
27	1.55	95321.42	5.21	anaphase promoting complex subunit 2
28	6.39	31393.08	9.04	killer cell lectin-like receptor, subfamily A, member 8
